# Adolescent and non-consensual anal sexual debut among Chinese men who have sex with men: a cross-sectional study

**DOI:** 10.1186/s12879-020-05466-w

**Published:** 2020-10-07

**Authors:** Weiming Tang, Yehua Wang, Wenting Huang, Dan Wu, Fan Yang, Yongshi Xu, Jason J. Ong, Hongyun Fu, Bin Yang, Cheng Wang, Wei Ma, Chongyi Wei, Joseph D. Tucker

**Affiliations:** 1 Dermatology Hospital of Southern Medical University, Guangzhou, 510095 China; 2 University of North Carolina at Chapel Hill Project-China, Guangzhou, 510095 China; 3 Faculty of Infectious and Tropical Diseases, London School of Hygiene and Tropical Medicine, London, UK; 4 Central Clinical School, Monash University, Melbourne, Australia; 5 Division of Community Health and Research, Eastern Virginia Medical School, Norfolk, Virginia, USA; 6 School of Public Health, Southern Medical University, Guangzhou, China; 7 School of Public Health, Shandong University, Jinan, China; 8 Rutgers University, New Brunswick, NJ USA

**Keywords:** Behaviors, HIV, Men who have sex with men (MSM), Non-consensual sex, Prevalence, Sexual debut

## Abstract

**Background:**

Adolescent sexual debut and non-consensual sex have been linked to higher sexual risk and STI infection in adulthood among men who have sex with men (MSM) in high-income countries. This study aimed to examine adolescent and non-consensual anal sexual debut among Chinese MSM and to evaluate factors associated with adolescent sexual debut and non-consensual anal sex.

**Methods:**

A cross-sectional study was conducted recently among Chinese men assigned male sex at birth, ≥18 years old, and who had ever engaged in anal sex with a man. Participants answered questions regarding socio-demographics, condomless sex, age at anal sexual debut with a man, and whether the first anal sex was consensual. Factors associated with an adolescent sexual debut (< 18 years old) and non-consensual sex at sexual debut were evaluated. We defined adolescent sexual debut as having anal sex with another man at 17 years old or younger, and the participants were asked whether their first male-to-male anal sex was non-consensual.

**Results:**

Overall, 2031 eligible men completed the survey. The mean age of sexual debut was 20.7 (SD = 4.3) years old. 17.6% (358/2031) of men reported adolescent sexual debut, and 5.0% (101/2031) reported a non-consensual sexual debut. The adolescent sexual debut was associated with having more male sexual partners (adjusted OR 1.10, 95% CI 1.06–1.15) and condomless anal sex in the last three months (AOR = 1.71, 95% CI 1.34–2.18). MSM whose sexual debut was non-consensual were more likely to have condomless anal sex (AOR = 1.76, 95% CI 1.17–2.66), and to have reported an adolescent sexual debut (AOR = 2.72, 95% CI 1.75–4.21).

**Conclusions:**

Many Chinese MSM reported adolescent sexual debut and non-consensual sex, both of which are associated with sexual risk behaviors and drive STI transmission. These findings highlight the need for designing tailored interventions for MSM who experienced adolescent sexual debut and non-consensual sex at debut.

## Background

Adolescent sexual debut and non-consensual anal sex among men who have sex with men (MSM) may contribute to adulthood high-risk behaviors and sexually transmitted infection (STI) transmission [1, 2]. Previous research with MSM suggests that adolescent sexual debut is associated with riskier sexual behaviors such as more partners, group sex, and recreational drug use [1, 3]. Previous studies have also indicated people who experienced non-consensual sex, to have traumatic experiences that tend to increase their risk of HIV infection, alcohol abuse, and poor health-seeking behaviors [2, 4–6]. However, most research on sexual debut and non-consensual sex among MSM has been focused on high-income countries [1, 7], and hence less is known about these phenomena in low- and middle-income countries where social taboos regarding same-sex behaviors are strongly endorsed but with limited denunciation of non-consensual sex [3]. Though non-consensual sex in MSM has been explored, [8, 9] limited studies to date have assessed this issue in the context of adolescent sexual debut. The intersection of these two factors is important because trauma related to early sexual experiences can lead to high-risk sexual behaviors in adulthood [1, 3].

Due to social and cultural pressures of the local environment, MSM in China are often subject to stigma and social discrimination [10, 11]. As a result, they receive limited sexual health education from guardians or in schools, potentially leaving them with limited knowledge of safer sex practices [12, 13]. Without scientifically accurate knowledge, early sexual experiences of individuals may further impact their subsequent behaviors, and facilitate engaging in risk-taking behaviors, such as condomless sex and having more sexual partners. In addition, men who experienced adolescent or non-consensual sexual debut may also suffer from psychological problems that they are associated with adopting maladaptive coping behaviors (e.g., heavy drinking) linked to HIV/STI risk [2]. For example, a study of Latino MSM in the US found that those experiencing childhood sexual abuses were more likely to report depressive symptoms and heavy drinking [9]. However, the studies on adolescent sexual debut and non-consensual sex among Chinese MSM are very limited [3, 14], and knowing these may guide the designing of future sexual health education and interventions for Chinese MSM.

China provides a strong environment for this study. First, with the popularization of gay dating apps [15], researchers in China can easily reach MSM who were usually marginalized through online surveys, and these participants are willing to report sensitive personal information anonymously [16]. This may provide an opportunity to disclose information that they would not reveal in clinic-based surveys. Second, Chinese MSM tend to be stigmatized and discriminated, and lack sexual health education and awareness of health care [11]. Third, the social norms and attitudes toward sexuality among Chinese MSM have rapidly changed in the past decade, and Chinese MSM are more open to having sex at an earlier age [17, 18]. In addition, Chinese MSM are disproportionally impacted by HIV, while the HIV prevalence among MSM reached 8.0% in 2015 and has remained high since then, which is about 300–400 times the prevalence observed among the general population in China [19, 20]. In addition, extremely high HIV incidence (15.56/100 person-years) was also observed among Chinese gay men [21].

This study aimed to examine adolescent and non-consensual sexual debut among Chinese MSM and to evaluate factors associated with adolescent sexual debut and non-consensual anal sex.

## Methods

This study is a secondary analysis of baseline data from a stepped-wedge randomized controlled trial (RCT). The study evaluated the efficacy of a crowdsourced intervention in promoting HIV testing among MSM in eight Chinese cities [16], while only those who did not report HIV testing in the preceding three months in the cross-sectional survey were enrolled into the RCT.

### Study design and sampling methods

The nationwide, online, cross-sectional study was conducted by the University of North Carolina Project-China in July 2016. We collaborated with a gay partner seeking internet company (Blued) to recruit participants from eight cities in China (Guangzhou, Jiangmen, Shenzhen, and Zhuhai in Guangdong Province, Jinan, Jining, Qingdao and Yantai in Shandong Province, China). Blued is the largest MSM social networking mobile phone application (app) in China, with more than 40 million registers. MSM usually use this platform for socialization, seeking HIV care services, and dating. While most MSM in China uses this platform, the frequency users tend to be young, well-educated, and with high income.

For recruitment, banner advertisements linking to the online survey were sent to registered Blued users in the eight cities. Participants who clicked on the survey link were directed to the online survey hosted by Sojump (Shanghai, China, http://www.sojump.com), a widely used online survey platform in China [22]. The inclusion criteria included: being Chinese men assigned male sex at birth, at least 18 years of age, having ever had anal sex with another man (identified as MSM), and currently residing in one of the designated study cities. Eligible participants provided electronically written informed consent. An incentive of 50 RMB ($ 7USD) was provided to the participants as a compensation for the survey completion. Quality control was assured by using the skipping patterns in the survey platform, and participants were not allowed to go back in the survey to change their previous answers, thus this study does not have the issue of missing data. We followed a checklist for reporting results of Internet e-surveys (CHERRIES) throughout the process to improve the quality and reporting of our web survey.

### Measures

The anonymous online survey included measures on socio-demographics, sexual behaviors, HIV testing history, and HIV status from all participants. Socio-demographic information included age (as a continuous variable and further categorized into four groups: under 20, 20–29, 30–39 or 40 and above), marital status (never married, currently married, and divorced or widowed), education (high school or below, some college, college/bachelors, and masters or above), Household registration (Hukou, the hukou refers to China’s national household registration system; it was categorized into the sampling city, other cities in the sampling province, or other provinces in China), and annual income (≤ $1500 USD, $1501–3000, $3001–5000, $5001–8000, or more than $8000). Every participant was asked to self-report their sexual orientation (gay or bisexual), whether they disclosed their sexual orientation to non-male sexual partners (yes or no), and whether they self-identified as a transgender woman (yes or no).

Participants answered whether they had a stable male partner (yes or no), a casual male partner (yes or no), a stable female partner (yes or no), and a casual female partner (yes or no) in the last three months. We also asked the number of male partners they had in the last three months (continuous), whether they engaged in anal sex with a stable male partner in the last three months (yes or no), whether they engaged in anal sex with a casual male partner in the last three months (yes or no), whether they engaged in condomless sex with a man in the last three months (yes or no), and the type of the last partner (stable or casual partner).

Survey questions also covered HIV testing history. Participants answered questions on whether they had ever tested for HIV (yes or no) and the results from their last HIV test (positive, negative, or unknown).

With regard to male-to-male sexual debut, the participants were asked the age at which they first engaged in anal sex with a man. We defined adolescent sexual debut as having anal sex with another man at 17 years old or younger, as 18 years old was considered to be adult in China, and having sex before 18 was considered to have sex early in China [23]. In addition, the participants were asked whether their first male-to-male anal sex was non-consensual (coerced or unwilling to have sex with the partner).

### Statistical analysis

Descriptive analysis was used to present the distribution of socio-demographic characteristics and HIV-related behaviors of the participants. Univariate and multivariable logistic regressions were used to evaluate factors associated with the adolescent sexual debut and non-consensual anal sex at sexual debut, while adolescent sexual debut and non-consensual anal sex at sexual debut were treated as outcomes, to make the data analysis easier. Age, residence, educational level, and annual income were adjusted in the multivariable logistic regressions. All data analysis was completed using SAS 9.4 (SAS int. Cary, NC, USA).

## Results

### Socio-demographics and sex behaviors

Overall, all the 2031 MSM who finished the baseline survey were included in the current study. Their median age was 26.3 (SD = 6.3) years old, while about three-quarters of participants were less than 30 years old (75.8%). In addition, the majority of the participants had household registration in the two study provinces (68.0%), never married (85.4%), and had attended at least some college (66.6%) (Table 1).

**Table 1:** Socio-demographic characteristics of men who have sex with men (MSM) and transgender individuals in China, 2016 (*N* = 2031)

Variables	Frequency	Percent (%)
**Age group (years)**		
*< 20*	185	9.1
*20–29*	1355	66.7
*30–39*	397	19.6
*≥ 40*	94	4.6
**Household registration (Hukou)**		
*The sampling city*	627	30.9
*Other cities in the sampling province*	754	37.1
*Other provinces*	650	32.0
**Marital Status**		
*Never married*	1735	85.4
*Currently married*	187	9.2
*Divorced or widowed*	109	5.4
**Education**		
*High school or below*	679	33.4
*Some college*	578	28.5
*College/Bachelors*	695	34.2
*Masters or above*	79	3.9
**Annual income (USD)**		
*= < 1500*	337	16.6
*1501–3000*	409	20.1
*3001–5000*	687	33.8
*5001–8000*	383	18.9
*= > 8001*	215	10.6
**Self-identified as transgender individuals**		
*Yes*	104	5.1
*No*	1927	94.9
**Sexual orientation**		
*Gay*	1473	72.5
*Bisexual*	558	27.5
**HIV status**		
*Living with HIV*	62	3.1
*At the risk of HIV*	986	48.5
*Unknown*	61	3.0
*Never tested*	922	45.4
**Sexual orientation disclosure**		
*Disclosed*	1381	68.0
*Non-disclosed*	650	32.0
**Age at sexual debut**		
*< 18 years old*	358	17.6
*18 years old or elder*	1673	82.4
**Non-consensual sex at sexual debut** ^a^		
*No*	1930	95.0
*Yes*	101	5.0

*Note:* ^a^ We defined adolescent sexual debut as having anal sex with another man at 17 years old or younger, and the participants were asked whether their first male-to-male anal sex was non-consensual

About 5% of the participants (5.1%) identified as transgender women. Close to three-quarters of the participants self-identified themselves as gay (72.5%), and about two-thirds of the participants reported that they ever disclosed their sexual orientation to others beyond their partners (68.0%). Overall, two-thirds of the participants reported having a regular male partner in the last three months (66.4%), and about two-fifths of the participants reported having a casual male partner in the last three months (40.7%).

Over 60% of the participants self-reported that they had ever tested for HIV (63.9%). About half of the participants reported that they did not know their HIV status (48.4%, either never tested before or did not get a test result). Of those who ever tested for HIV, 5.9% reported that they were living with HIV (62/1048).

### Sexual debut and non-consensual sex

The median and mean ages of reported sexual debut were close, so we only reported mean age in the current study. Overall, 17.6% (358/2031) of men reported adolescent sexual debut. The mean ages at anal sexual debut for participants in the age groups of < 20, 20–29, 30–39 and 40 or above were 17.1 (SD = 1.4), 19.8 (SD = 2.8), 23.2 (SD = 4.4) and 29.7 (SD = 7.5) years old (*p* < 0.001 for each comparison), respectively.

In addition, 5.0% of participants self-reported that they experienced non-consensual sex at anal sexual debut (101/2031). The mean ages at anal sexual debut for participants who experienced and who did not experience non-consensual sex were 15.8 (SD = 1.5) and 21.8 (SD = 4.0) years old (*p* < 0.001), respectively. The proportion of participants experiencing non-consensual sex at anal sexual debut with another man was higher in those whose sexual debut happened before age 18 (10.1%), as compared to those for whom it happened at 18 or later (3.9%, *p* < 0.001) .

### Factors associated with adolescent sexual debut

Results from multivariable regression analysis revealed higher odds of adolescent sexual debut in men who disclosed sexual orientation to others (adjusted odds ratio [aOR], 1.84; 95% confidence interval [CI], 1.40–2.43) compared to men who did not disclose their sexual orientation. In addition, men reporting adolescent sexual debut with a male tended to have two or more sexual partners in the last three months (aOR = 1.10, 95% CI: 1.06–1.15), were more likely to engage in condomless anal sex with a male partner in the last three months (aOR = 1.71, 95% CI: 1.34–2.18), and to report that their most recent sexual partner being a casual partner (aOR = 1.29, 95% CI: 1.02–1.63) (Table 2).

**Table 2:** Factors associated with adolescent sexual debut among Chinese men who have sex with men (MSM), 2016 (*N* = 2031)

Variables	Crude OR (95% CI)	Adjusted OR (95% CI) ^a^
**Sexual Orientation**		
*Bisexual*	0.88 (0.58, 1.14)	0.97 (0.74, 1.27)
*Gay*	*Ref.*	
**Ever disclosed sexual orientation to others**		
*Yes*	**190 (1.45, 2.49)**	**1.84 (1.40, 2.43)**
*No*	*Ref.*	
**Transgender individuals**		
*No*	0.84 (0.51, 1.37)	0.84 (0.51, 1.40)
*Yes*	*Ref.*	
**Number of male partners in the last three months**	**1.09 (1.04, 1.14)**	**1.10 (1.06, 1.15)**
**Anal sex with stable male partners in 3 months**		
*Yes*	**1.49 (1.17, 1.89)**	**1.58 (1.24, 2.02)**
*No*	*Ref.*	
**Condomless anal sex in the past 3 months**		
*Yes*	**1.61 (1.27, 2.05)**	**1.71 (1.34, 2.18)**
*No*	*Ref.*	
**Anal sex with casual male partners in 3 months**		
*Yes*	**1.99 (1.15, 3.46)**	**2.06 (1.18, 3.59)**
*No*	*Ref.*	
**Stable or casual partner as your last sex partner**		
*Casual partner*	**1.29 (1.03,1.63)**	**1.29 (1.02,1.63)**
*Stable partner*	*Ref.*	
**Ever tested for HIV**		
*Yes*	0.99 (0.78, 1.26)	1.08 (0.85, 1.38)
*No*	*Ref.*	
**HIV status**^b^		
*Living with HIV*	1.36 (0.73, 2.52)	1.30 (0.69, 2.46)
*Unknown*	0.60 (0.27, 1.35)	0.59 (0.26, 1.34)
*At the risk of HIV*	*Ref.*	

*Note* ^a^*Models were adjusted for participants’ marital status, household registration, and education level. Due to high collinearity, age was not adjusted;* ^b^*among people who ever tested for HIV*

### Factors associated with non-consensual sex at sexual debut with a male

Results from multivariable logistic regression model demonstrated that men who reported non-consensual sex at sexual debut had higher odds of reporting adolescent sexual debut (aOR = 2.72, 95% CI: 1.76–4.21) and engaged in condomless anal sex with a male partner in the last three months (aOR = 1.76, 95% CI: 1.17–2.66). In addition, men whose sexual debut with a male was non-consensual were more likely to have ever tested for HIV (aOR = 1.56, 95% CI: 1.01–2.42) (Table 3).

**Table 3:** Factors associated with non-consensual sex at sexual debut among Chinese men who have sex with men (MSM), 2016 (*N* = 2031)

Variables	Crude OR (95% CI)	Adjusted OR (95% CI) ^a^
Sexual orientation		
*Bisexual*	**1.12 (0.72,1.74)**	**1.10 (0.70,1.72)**
*Gay*	*Ref.*	
Ever disclosed sexual orientation to others		
*Yes*	1.07 (0.69,1.65)	1.11 (0.72,1.73)
*No*	*Ref.*	
Transgender individuals		
*No*	1.04 (0.41,2.61)	1.04 (0.41,2.63)
*Yes*	*Ref.*	
Adolescent sexual debut		
*Yes*	**2.77 (1.81,4.23)**	**2.72 (1.76, 4.21)**
*No*	*Ref.*	
Number of male partners in last 3 months	1.00 (0.96,1.04)	1.00 (0.97,1.04)
Anal sex with stable male partners in 3 months		
*Yes*	0.78 (0.46,1.30)	0.78 (0.46,1.31)
*No*	*Ref.*	
Condomless anal sex in 3 months		
*Yes*	**1.70 (1.13,2.57)**	**1.76 (1.17,2.66)**
*No*	*Ref.*	
Anal sex with casual male partners in 3 months		
*No*	0.77 (0.37, 1.59)	0.80 (0.39, 1.67)
*Yes*	*Ref.*	
Stable or casual partner as your last sex partner		
*Stable partner*	0.70 (0.47,1.04)	080 (0.47,1.05)
*Casual partner*	*Ref.*	
Ever tested for HIV		
*Yes*	1.43 (0.92,2.22)	**1.56 (1.01,2.42)**
*No*	*Ref.*	
HIV status^b^		
*Living with HIV*	1.44 (0.50, 4.15)	1.33 (0.46, 3.88)
*Unknown*	**3.16 (1.42, 7.03)**	**2.97 (1.33, 6.65)**
*At the risk of HIV*	*Ref.*	

*Note* ^a^*Models were adjusted for participants’ marital status, household registration, and education level. Due to high collinearity, age was not adjusted;* ^b^*among people who ever tested for HIV*

## Discussion

Adolescent sexual debut and non-consensual sex at sexual debut with a male were closely correlated with subsequent condomless anal sex and among Chinese MSM. Our findings indicate that 5% of MSM in the study experienced a non-consensual sexual debut. This study adds to the current literature by providing data on sexual debut and non-consensual sex at sexual debut with a male and identifying the correlates of adolescent sexual debut and non-consensual sex among Chinese MSM, and it is a rare example of research on adolescent and non-consensual anal sexual debut among MSM in a low- and middle- income countries (LMICs) context.

We found that about 18% of the participants’ experienced adolescent anal sexual debut, and at the mean age of 20.7 years old. The mean age at anal sexual debut among the participants in our study was similar to two studies in China, [3, 14] However, the mean ages of debut in this study were older than those reported among MSM in the US [5]. Similar to a study conducted in China in 2012–2013, our study also indicated that the age at sexual debut for Chinese MSM were lower among younger participants, which may partially explain the younger age of debut observed in our study. The decreasing age at anal sexual debut may reflect the sexual revolution and changing pattern for partner seeking among MSM in China. Before 2008, the main settings where Chinese MSM identified male partners were public parks, bars, or bathhouse [24]. Considering that many Chinese MSM reported discomfort with visiting gay bars or bathhouses due to fears of being identified in public as a gay man, [25] challenges identifying potential male partners may have delayed their age at sexual debut. However, the rise in popularity of the internet and social media (i.e., gay dating apps) sites for MSM in China has reduced challenges associated with sexual partner seeking, potentially facilitating sex initiation at earlier ages. In addition, as a result of rapid cultural change, social norms about sex among Chinese MSM have also rapidly changed in the past decade, which may have also made MSM more likely to initiate male-to-male anal sex early. We therefore recommend that clinicians should screen for factors like adolescent sexual debut and/or coercive sex when providing HIV services to Chinese MSM.

We found that about 5% of the participants reported a non-consensual anal sexual debut. The proportion of non-consensual anal sex in our study was lower than in a previous small study from China [14]. This proportion was much lower than observed by studies conducted in Ecuador (26.7%) [26] and Myanmar (15%) [27]. The limited literature on non-consensual sex among MSM also indicated that a higher proportion of MSM experienced non-consensual sex than other men and those who experienced non-consensual sex usually suffer a range of long-term health effects [2, 28]. Previous studies have reported that MSM who experienced sexual abuse were more likely to later report depression, problematic alcohol use, more partners, more frequent condomless sex, and higher HIV prevalence [9, 29], and hence, on-time supportive services to help them overcome these problems are needed. Further implementation studies that aim to provide and evaluate the effect of supportive services for the victims are also needed. In addition, hotlines and online help queries should also be set up in order to provide timely supportive services to victims.

We also found that about 10% of men with adolescent anal sexual debut also reported non-consensual anal sex. Due to the limited literature on this topic, we did not find any similar study on this topic. However, the strong association between adolescent sexual debut and non-consensual sex in our study suggests that some young gay men are being assaulted or coerced. One potential reason for this phenomenon is that there is a lack of legal protection for gay men, and in the event of a same-sex assault, the perpetrators were rarely held accountable, and victims remain unprotected in China. Thus, strategies such as providing community support and protective services (i.e., anonymous reporting platforms and support groups) to people who experienced non-consensual sexual debut would be important. Legal aid and psychological counseling would also be useful, especially in China where the current criminal law does not recognize men being victims of rape [30].

We found that men who reported both adolescent and non-consensual sexual debut were more likely to engage in condomless anal sex. This finding is consistent with the limited literature on adolescent sexual debut [7, 14, 17, 31]. The script theory and life-course models indicate that the early sexual experience of a man could shape their preferences of the characteristics of certain partners, and impact their subsequent sexual behavior with different partners [14, 32]. Another potential explanation for this phenomenon is that both adolescent and non-consensual sexual debut can lead to psychological problems and self-harm, while these issues are highly correlated with condomless sex. [33, 34] However, we did not collect this information in our study, and further studies on understanding the mechanism of this phenomenon among Chinese MSM are needed. Approaches for promoting condom use during and after sexual debut among MSM would be essential.

Our study has several limitations. First, the cross-sectional nature of this study means that all the correlations identified in this study should be inferred with caution. Second, we did not collect information on violence, mental health, alcohol, and substance abuse from the participants, while these variables are considered to be highly correlated with adolescent sexual debut and non-consensual sex. Third, as an online survey conducted in eight Chinese cities, our results cannot represent the general situation among Chinese MSM, as the online recruited participants tended to be young and well-educated. Fourth, as all data collected were self-reported, and since same-sex sexual activity is stigmatized in China, the rates of adolescent sexual debut and coerced sex may be underestimated in China, and social desirability bias may be present. However, we anticipate that this bias is small since the survey was online and no face-to-face meeting was involved. Regardless, our study provided useful information for a glimpse of the whole picture of adolescent sexual debut and non-consensual sex among Chinese MSM.

## Conclusion

A substantial proportion of Chinese MSM reported adolescent anal sexual debut, and 5% of MSM reported non-consensual anal sex at sexual debut. In addition, both adolescent sexual debut and non-consensual sex were associated with current risk behaviors such as condomless sex. These findings highlight the need for evidence-based sex education and intervention to protect the sexual autonomy of young men in the rising male-to-male sex culture in contemporary China. The findings of this study also have implications for expanding research about sexuality among youth. For example, studies evaluating the lifetime and recent non-consensual sex events, law or legal support needs, and health service needs would be very useful for providing tailored services for MSM.

## Data Availability

The datasets used and/or analyzed during the current study are available from the corresponding author on reasonable request.

## Abbreviations

CI: Confidence interval
MSM: Who have sex with men
LMIC: Low- and middle- income countries
RCT: Randomized controlled trial
STI: Sexually transmitted infection
